# Gold Nanoparticle-Based Plasmonic Detection of *Escherichia coli*, *Salmonella enterica*, *Campylobacter jejuni,* and *Listeria monocytogenes* from Bovine Fecal Samples

**DOI:** 10.3390/microorganisms12061069

**Published:** 2024-05-25

**Authors:** Ahmed Ghazy, Rejoice Nyarku, Rawah Faraj, Kingsley Bentum, Yilkal Woube, McCoy Williams, Evangelyn Alocilja, Woubit Abebe

**Affiliations:** 1 Veterinary Services Department of Egyptian Armed Forces, Cairo 11768, Egypt; ahmed.ghazy366@gmail.com; 2 Food Hygiene and Control Department, Faculty of Veterinary Medicine, Sadat University, Sadat City 32897, Egypt; 3 Center for Food Animal Health, Food Safety, and Food Defense, Department of Pathobiology, College of Veterinary Medicine, Tuskegee University, Tuskegee, AL 36088, USA; rnyarku8794@tuskegee.edu (R.N.); rfaraj@tuskegee.edu (R.F.); kbentum8786@tuskegee.edu (K.B.); ywoube@tuskegee.edu (Y.W.); mwilliams1720@tuskegee.edu (M.W.); 4 Biosystems and Agricultural Engineering, Michigan State University, East Lansing, MI 48824, USA; alocilja@msu.edu; 5 Global Alliance for Rapid Diagnostics, Michigan State University, East Lansing, MI 48824, USA

**Keywords:** plasmonic biosensor, magnetic nanoparticles, gold nanoparticles, fecal samples, *Salmonella*, limit of detection (LOD)

## Abstract

Current diagnostic methods for detecting foodborne pathogens are time-consuming, require sophisticated equipment, and have a low specificity and sensitivity. Magnetic nanoparticles (MNPs) and plasmonic/colorimetric biosensors like gold nanoparticles (GNPs) are cost-effective, high-throughput, precise, and rapid. This study aimed to validate the use of MNPs and GNPs for the early detection of *Escherichia coli* O157:H7, *Salmonella enterica* spp., *Campylobacter jejuni*, and *Listeria monocytogenes* in bovine fecal samples. The capture efficiency (CE) of the MNPs was determined by using *Salmonella* Typhimurium (ATCC_13311) adjusted at an original concentration of 1.5 × 10^8^ CFU/mL. One (1) mL of this bacterial suspension was spiked into bovine fecal suspension (1 g of fecal sample in 9 mL PBS) and serially diluted ten-fold. DNA was extracted from *Salmonella* Typhimurium to determine the analytical specificity and sensitivity/LOD of the GNPs. The results showed that the CE of the MNPs ranged from 99% to 100% and could capture as little as 1 CFU/mL. The LOD of the GNPs biosensor was 2.9 µg/µL. The GNPs biosensor was also tested on DNA from 38 naturally obtained bovine fecal samples. Out of the 38 fecal samples tested, 81.6% (31/38) were positive for *Salmonella enterica* spp., 65.8% (25/38) for *C. jejuni*, 55.3% (21/38) for *L. monocytogenes*, and 50% (19/38) for *E. coli* O157:H7. We have demonstrated that MNP and GNP biosensors can detect pathogens or their DNA at low concentrations. Ensuring food safety throughout the supply chain is paramount, given that these pathogens may be present in cattle feces and contaminate beef during slaughter.

## 1. Introduction

Foodborne pathogens pose serious threats to food safety and result in human diseases when pathogens or their toxin-contaminated animal products are consumed [1]. Foodborne pathogens have increased in importance as a global public health issue in recent years, and their effects on economics and health (a high morbidity and mortality rate) are now well acknowledged [2]. The main reservoirs for many foodborne infections are animals raised for food, such as cattle, chickens, pigs, and turkeys [1].

Shiga toxin-producing *Escherichia coli*, *Salmonella enterica* spp., *Campylobacter jejuni*, and *Listeria monocytogenes* are the top four foodborne bacterial pathogens in the United States and Europe [3,4,5]. Infection with *L. monocytogenes* is associated with abortions in both humans and animals, with fever, headaches, nausea, vomiting, abdominal pain, and diarrhea. Salmonellosis outbreaks have been linked to foods, including eggs, poultry, and other items of animal origin. *Campylobacter* foodborne cases primarily occur through consuming contaminated foods, such as raw milk and raw or undercooked chicken, and drinking contaminated water. Unpasteurized milk, undercooked meat, and contaminated fresh produce are all linked to enterohemorrhagic *E. coli* [6]. Due to the significant impact of foodborne pathogens on public health, it is imperative to implement effective detection methods for these pathogens [7,8].

Existing detection methods include culture-based, molecular, and immunoassay techniques, which are labor-intensive and time-consuming [9]. Despite numerous advancements in the diagnostic sector, it remains challenging to identify novel strategies that have an enhanced simplicity, specificity, stability, and sensitivity [10]. High specificity and quick assay times (1.5 to 2 h) have been achieved with immunoassays and molecular techniques like polymerase chain reaction (PCR). However, background microbiota and dietary matrices may interact with or hinder specific immunoassays and PCR assays [11]. Additionally, it is challenging to include immunoassays into an array to detect numerous targets, due to the significant cross-reactivity of antibodies [11].

The development of biosensors has yielded encouraging results, making them one of the most practical and user-friendly on-site detection technologies [12], due to their ease of use, the possibility for downsizing, and the capability for real-time analysis [13]. Biosensors outperform standard laboratory assays in terms of sensitivity, specificity, accuracy, speed of response, and potential portability. They are now considered promising alternative tools and one of the most site-applicable and user-friendly fast detection approaches [12].

Optical biosensors have a transducer that can convert the interactions between receptors and their targets into quantifiable light signals [14]. Based on various optical signal-transducing methods, they are primarily divided into four types: colorimetric/plasmonic, surface-enhanced Raman scattering (SERS), fluorescent, and surface plasmon resonance (SPR) biosensors [14]. Among these, colorimetric/plasmonic techniques are used for qualitative and semiquantitative screening without complex apparatus because the signals produced may be seen with the naked eye [12]. Plasmonic detection is commonly employed for DNA detection [15], and due to its simplicity, sensitivity, stability, and quickness, it has garnered increased interest [16].

**Figure 1:**
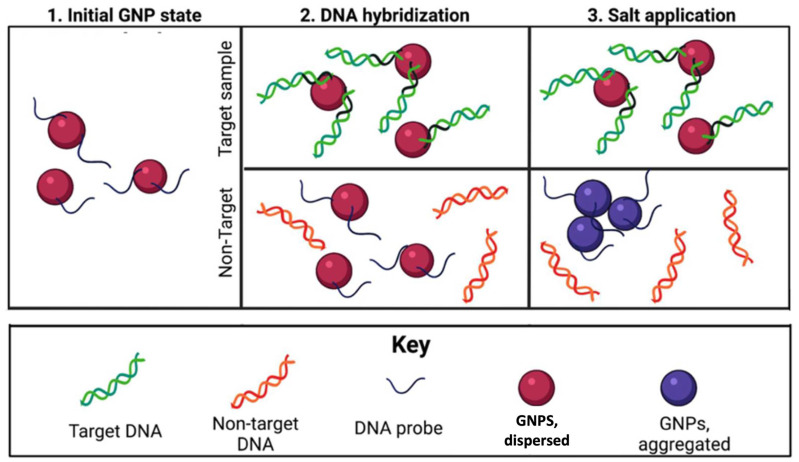
A general mechanism for target-aggregating *G*NP plasmonic/colorimetric DNA biosensors. Image from [19].

Nanomaterials are frequently integrated into the design of optical biosensors to increase their sensitivity [17]. Gold nanoparticles (GNPs) emit a dynamic spectrum of absorption peaks, reacting to varying target concentrations [18]. Upon aggregation, the GNPs’ size changes, the solution’s color shifts to blue/purple, and the absorption peak at high wavelengths becomes more prominent. On the other hand, when dispersed in solution, GNPs are ruby red [19,20,21], which is illustrated in (Figure 1).

The MNP and GNP biosensors used in this study have already been validated in similar experiments using other organisms and food matrices [20,22,23,24]. However, the performance of the biosensor in bovine fecal samples has not yet been explored. Cattle carry the most abundant bacterial zoonoses known to humans. Among these are four priority pathogens, a source of pre-and post-harvest food contamination [1]. Ensuring food safety throughout the supply chain is paramount, given that these pathogens may be present in cattle feces and contaminate beef during slaughter.

Hence, this study aims to validate two distinct methodologies (the use of MNPs and GNPs) to identify the presence of four priority foodborne pathogens. Specifically, we strive to adopt the use of MNPs to capture/concentrate bacteria for culture isolation and the use of plasmonic/colorimetric GNPs biosensors for the early detection of *E. coli* O157:H7, *Salmonella enterica* spp., *C. jejuni*, and *L. monocytogenes* DNA from a complex bovine fecal sample. Ultimately, the objective is to allow early detection and mitigate the further spread of these pathogens to the environment, the food chain, and humans. Therefore, this study determines the capture efficiency of the MNPs, determines the analytical specificity and sensitivity/limit of detection (LOD) of the GNPs plasmonic/colorimetric biosensor, and finally, evaluates the performance of the GNPs biosensor on naturally contaminated bovine fecal samples.

## 2. Materials and Methods

### 2.1. Reagents and Chemicals

The Alocilja Research Group from Michigan State University (East Lansing, MI, USA) supplied the glycan-coated magnetic nanoparticles (MNPs) and gold nanoparticles (GNPs), and their mechanisms of action are briefly described. Briefly, glycan-coated magnetic nanoparticles (MNPs) are manufactured with a core made of iron oxide (III) or magnetite (Fe_3_O_4_) and a shell made of glycan (chitosan). The synthesis of Fe_3_O_4_ involves the utilization of ferric chloride hexahydrate (FeCl_3_·6H_2_O) as a precursor within a solution containing ethylene glycol as a reducing agent and sodium acetate as a porogen. Polymerization is then employed to modify the surface of the iron oxide nanoparticles using chitosan. The glycan coating on the magnetic nanoparticles (MNPs) facilitates the cost-effective and straightforward capture of bacterial cells, achieved through the interaction between the glycan on the MNP and the glycoproteins on the surface of the bacterial cells. This eliminates the need for costly antibodies or aptamers in immunomagnetic separation techniques [25].

### 2.2. Bacterial Culture

American Type Culture Collection (ATCC) strains of *Salmonella* Typhimurium, *E. coli* O157:H7, *C. jejuni*, and *L. monocytogenes* were obtained from the Center of Food Safety Laboratory, Tuskegee University, Tuskegee, AL, USA. Each bacterial strain was cultured in Tryptic soy broth primary enrichments at 37 °C for 24 h.

### 2.3. Spiking of Bovine Fecal Samples Using Salmonella as a Model

*Salmonella* Typhimurium (ATCC_13311) was cultured in Tryptic soy broth at 37 °C for 24 h and centrifuged to pellet bacterial cells. The supernatant was discarded, and the bacterial pellets were resuspended in 1× phosphate buffer saline (PBS) to obtain a 0.5 McFarland standard concentration (1.5 × 10^8^ CFU/mL) with a 0.1 absorbance using a turbidimeter. After this, 1 mL of the 0.5 McFarland standard was spiked into 9 mL of bovine fecal suspension (1 g of fecal sample in 9 mL PBS). The fecal sample had been confirmed through molecular testing and culture as free of the four pathogens. A ten-fold serial dilution of the bacteria-spiked stock sample was prepared from 10^−1^ to 10^−7^; thus, 100 µL of stock into 900 µL PBS in seven Eppendorf tubes. The dilution at 10^−4^, the fourth dilution factor corresponding to 1.5 × 10^4^ CFU/mL, was used as the starting stock solution for the experiment and denoted as 10^−1^. Those steps mentioned above were carried out into two different sets. One set (set A) was for the MNP experiment (with the addition of MNPs), and another group (set B) was for direct plating (no MNPs were added to the sample); this was for colony enumeration.

### 2.4. Capture Efficiency of MNPs

The MNP experiment (set A) was carried out as described by [22] with some modifications. Briefly, a volume of ten microliters (10 µL) of 5 mg/mL MNP concentration was pipetted into each tube, containing serially diluted spiked fecal samples ranging from 10^−1^ (1.5 × 10^4^ CFU/mL) to 10^−5^ (1.5 CFU/mL) dilutions. The tubes were kept in a shaker incubator at a temperature of 32 °C for 10 min, after which they were placed on a magnetic rack for 5 min to pull the MNPs–bacteria to the side of the magnetic field, allowing for the remaining fluid to be drawn out and discarded without disturbing the captured MNPs bound to the bacteria. The MNP–bacteria complex was resuspended in 90 µL of 1× phosphate-buffered saline (PBS). Later, the resuspension volume of 90 µL was inoculated onto Xylose Lysine Deoxycholate (XLD) agar and incubated at 37 °C for 24 h.

To determine the number of colonies for each dilution (set B), 20 µL of each dilution series was directly plated on XLD agar and incubated at 37 °C for 24 h to determine the colony-forming units per milliliter (CFU/mL) in each dilution. The colonies on each plate for all serial dilutions (including sets A and B) were enumerated. The capture efficiency (CE) was therefore determined by dividing the log10 of the number of cells captured by the log10 of the number of cells before capture [22]:

$$CE\;\left(\%\right)=\frac{Log10\;\left(captured\;cells\;CFU/mL\right))}{\mathrm{Log}10\;\left(cell\;count\;before\;capture\;CFU/mL\right)}\times 100\;$$

The MNP experiment was carried out in two trials, and the mean colony count was used to calculate the capture efficiency.

### 2.5. Analytical Specificity of GNPs Biosensor in Detecting Salmonella enterica spp.

*Salmonella* Typhimurium (ATCC_13311) was cultured in Tryptic soy broth at 37 °C for 24 h and centrifuged to pellet bacterial cells [23]. DNA was extracted using the DNeasy Blood & Tissue Kit (Qiagen, Germantown, MD, USA) according to the manufacturer’s instructions. Subsequently, the extracted DNA was used in the GNPs biosensor experiment. Following the above procedure, the *E. coli* O157:H7, *L. monocytogenes*, and *C. jejuni* ATCC strains were isolated and subjected to DNA extraction, and these were used as non-target controls to assess the assay’s specificity. The experiment also included a mixed DNA comprising 5 µL *Salmonella* Typhimurium DNA and 5 µL *L. monocytogenes* DNA and the target DNA known as *Salmonella* Typhimurium.

The protocol for specificity testing was carried out as described by Dester et al. [19]. Briefly, 5 µL of GNPs was added to a 5 µL probe and 10 µL of DNA in a 0.2 mL tube. All the tubes were placed in a thermocycler for hybridization. The thermocycling conditions included a single cycle with a denaturing phase set at 95 °C for 5 min, an annealing phase at 55 °C for 10 min, and cooling at 4 °C for 5 min. Three microliters (3 μL) of 0.1 M HCL was added gradually to each tube, and the results were read after 5–10 min. Non-target DNA aggregated and turned purple/blue, while the target DNA remained red. A NanoDrop™ 2000 C spectrophotometer (Thermo Scientific™, Hanover Park, IL, USA) was used to read the absorbance of DNA at 520 nm.

**Figure 2:**
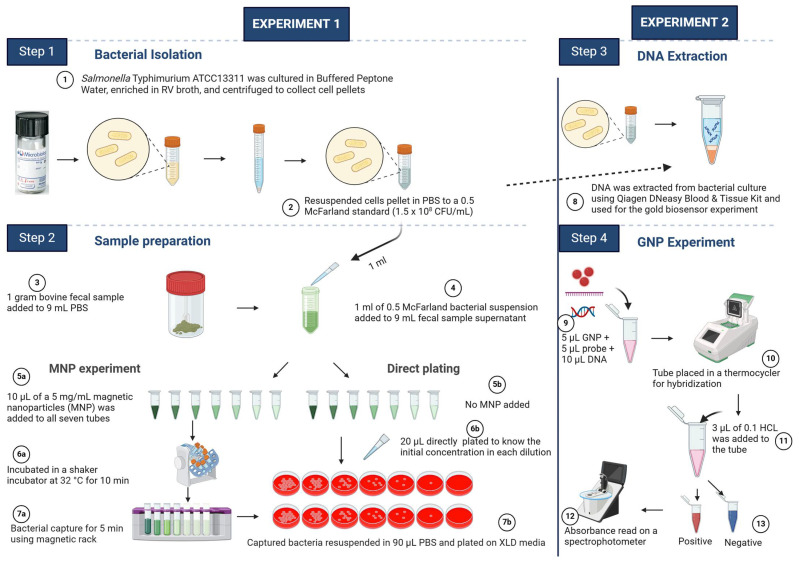
Schematic illustration of the steps used in the MNP capture of *Salmonella* Typhimurium from bovine fecal samples and the use of a GNPs plasmonic biosensor for the detection of *E. coli* O157:H7, *S. enterica* spp., *C. jejuni*, and *L. monocytogenes* from bovine fecal samples. The image was created with Biorender.com (Accessed on 10 May 2024).

The bacterial strains and probes used for the study are listed in Table 1 and Table 2, respectively. All the procedures described in Section 2.3, Section 2.4 and Section 2.5 are schematically represented in Figure 2.

### 2.6. Analytical Sensitivity of GNPs Biosensor in Detecting Salmonella enterica spp.

To determine the limit of detection (LOD) of the GNPs biosensor, *Salmonella* Typhimurium (ATCC_13311) was cultured in Tryptic soy broth at 37 °C for 24 h and centrifuged to pellet bacterial cells [26]. DNA was extracted using the DNeasy Blood & Tissue Kit (Qiagen, San Diego, CA, USA) according to the manufacturer’s instructions. The DNA concentration was measured and diluted to a 54.2 ng/µL concentration. A 1:2 dilution series was carried out from 2^−1^ to 2^−7^ and then tested with a GNPs biosensor following steps recommended by [20]. The experiment was conducted in five trials with each run performed in duplicates. A UV–Vis Spectra-NanoDrop™ 2000C spectrophotometer (Thermo Scientific™, Hanover Park, IL, USA) was used to measure the fluorescents produced by the reaction resulting in the color change, and the generated values were subjected to a two-way ANOVA statistical test using GraphPad Prism 10.0.3. The statistical analysis used a 95% confidence interval of wavelengths corresponding to the peak absorbance of targets at 520 nm to compare to non-targets above 600 nm.

### 2.7. Analysis of Field Bovine Fecal Samples

DNA extracted from thirty-eight bovine fecal samples from eighteen cattle farms in Alabama, USA, were obtained from the Center for Food Animal Health, Food Safety, and Food Defense Laboratory, Tuskegee University, Tuskegee, AL, USA. The status of these samples (positive or negative for all four pathogens) has been validated in parallel projects in our laboratory using PCR and culture. These samples were tested to determine the performance of the GNPs biosensor, based on the procedure described in Section 2.5.

## 3. Results

### 3.1. Capture Efficiency of MNPs

**Figure 3:**
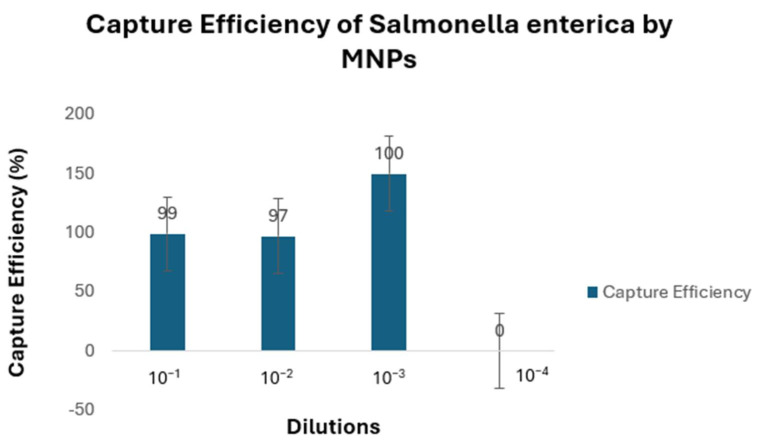
Capture efficiency of *Salmonella enterica* by glycan-coated MNPs. The numbers above the columns are the log10 of the number of cells captured by the MNPs (CFU/mL) divided by the number of cells in the initial dilution (CFU/mL), expressed as a percentage.

**Figure 4:**
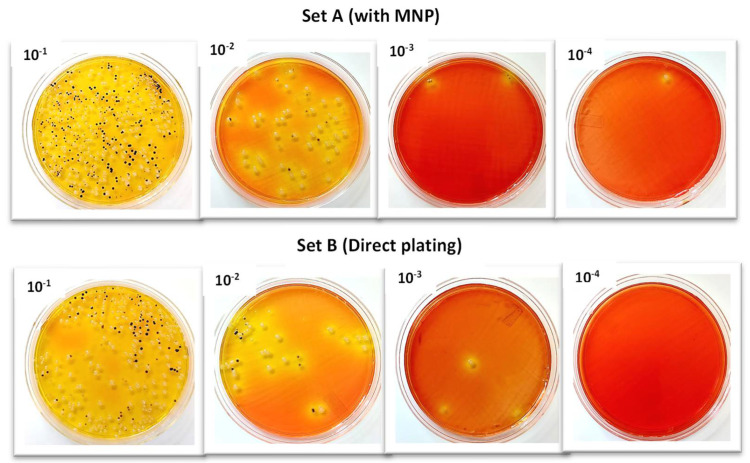
Capture of *Salmonella enterica* on XLD agar; (Set A) with MNPs and (Set B) direct plating.

The results indicated that the CE of the MNPs ranged from 99% to 100% (Figure 3). The CE at 10^−1^ was 99% at a concentration of 1.5 × 10^4^ CFU/mL. The MNPs captured Salmonella up to 1 CFU (Figure 4).

### 3.2. Analytical Specificity of GNPs Biosensor in Detecting Salmonella enterica spp.

**Figure 5:**
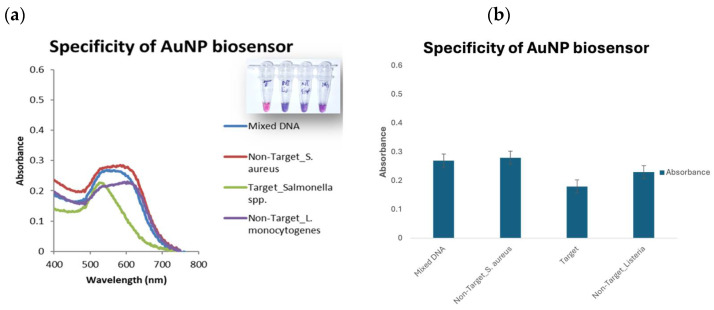
Specificity of GNPs biosensor. Bacterial pathogens include *Salmonella enterica* spp. (target); *L. monocytogenes* and *S. aureus* are non-targets, and their DNA is used in mixed DNA. Insert image is corresponding plasmonic image. (**a**) UV–Vis spectrum and (**b**) graph showing absorbance at 520 nm.

DNA was extracted from all four pathogens and used for specificity testing. The results were read using the color change with the naked eye and the UV–Vis spectrophotometer. The GNP biosensor detected the target DNA in the presence of closely related species, eliminating false positives and negatives (Figure 5). The aggregation of the GNPs will result in an increased peak wavelength absorption (about 600 nm or higher) and an apparent color change to blue or purple (negative result). In contrast, small and dispersed GNPs will have a peak absorbance of around 520 nm and appear red (positive result) [20].

### 3.3. Analytical Sensitivity of GNPs Biosensor in Detecting Salmonella enterica spp.

**Figure 6:**
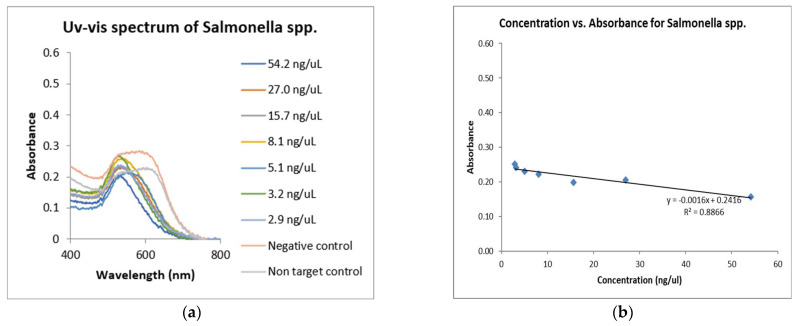
Sensitivity of GNPs biosensor. (**a**) represents plasmonic signals with varying DNA concentrations of *Salmonella* spp. The negative control is the non-spiked fecal sample, and the non-target control is *L. monocytogenes* DNA. (**b**) represents the calibration curves of the DNA concentration with their corresponding absorbance at 520 nm. The two-way ANOVA result shows a statistical significance between the absorbance for each dilution and between non-targets and targets; *p*-value < 0.0001 with an alpha level set to 0.05.

The performance of the GNPs biosensor was investigated (Figure 6). The linear regression equation was y = −0.0016x + 0.2416 (R^2^ = 0.8866). The detection limit (LOD) of the GNPs biosensor was 2.9 µg/µL. There was a statistical significance between the absorbance for each dilution and between non-targets and targets; *p*-value < 0.0001 with an alpha level set to 0.05.

### 3.4. Analysis of Field Bovine Fecal Samples

**Figure 7:**
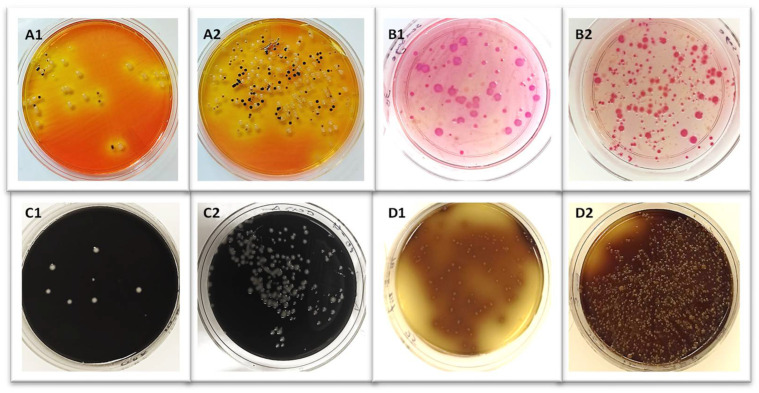
Bacterial colonies from bovine fecal samples captured by MNP. (**A1**,**B1**,**C1**,**D1**) indicate colonies without MNPs capture. (**A2**,**B2**,**C2**,**D2**) show bacterial colonies with MNPs capture for *Salmonella enterica* spp. (**A**); *E. coli* O157:H7 (**B**); *C. jejuni* (**C**); and *L. monocytogenes* (**D**).

**Figure 8:**
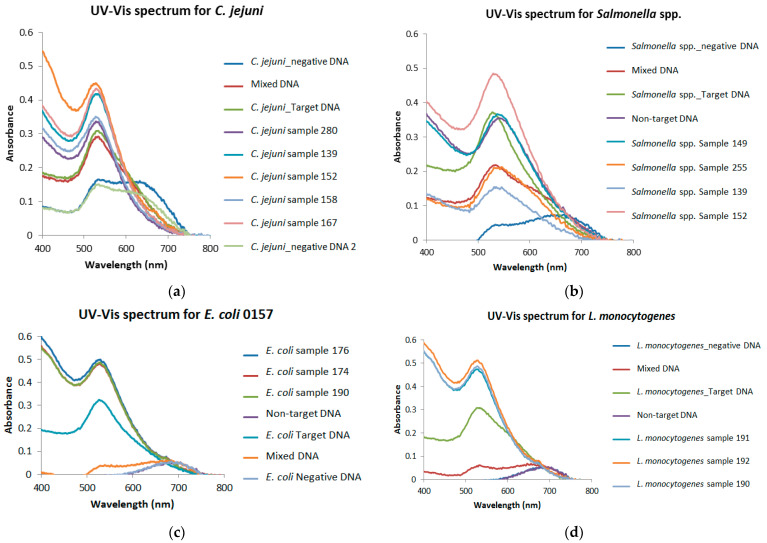
GNPs biosensor performance on bovine fecal samples in detecting *C. jejuni* (**a**), *Salmonella* spp. (**b**), *E. coli* O157:H7 (**c**), and *L. monocytogenes* (**d**).

The GNPs’ bacterial cell capture capability was also tested on these naturally infected fecal samples (Figure 7). Out of the thirty-eight (38) samples isolated with GNPs, 81.6% (31/38) tested positive for *Salmonella enterica* spp., 65.8% (25/38) tested positive for *C. jejuni*, 55.3% (21/38) tested positive *for L. monocytogenes*, and 50% (19/) tested positive for *E. coli* O157:H7 (Figure 8 and Table 3 and Table 4).

## 4. Discussion

This study aimed to validate two distinct methodologies (the use of MNPs and GNPs) to identify the presence of four priority foodborne pathogens. Specifically, it aimed to adopt the use of MNPs to capture/concentrate bacteria for culture isolation and the use of plasmonic/colorimetric GNPs biosensors for the early detection of *E. coli* O157:H7, *Salmonella enterica* spp., *C. jejuni*, and *L. monocytogenes* DNA from complex bovine fecal samples. The Interagency Food Safety Analytics Collaboration (IFSAC) named these four priority pathogens due to the significant number of outbreaks they cause collectively each year in the United States. Therefore, a tool that allows for the early detection of carrier animals will help mitigate further dissemination and curtail pre-and post-harvest food contamination. Hence, we determined the capture efficiency of the MNPs and the analytical specificity and the sensitivity/limit of detection (LOD) of the GNPs plasmonic/colorimetric biosensor for the four priority pathogens. We have also determined the performance of the GNPs biosensor directly from naturally contaminated bovine fecal samples.

This study used *Salmonella* Typhimurium (ATCC13311) as a model organism to determine the CE of MNPs. The CE ranged from 99% to 100%. The CE at 10^−1^ was 99% at a concentration of 1.52 × 10^4^ CFU/mL. The inverse correlation between bacterial cell concentration and the CE suggests that MNPs might be particularly valuable in situations with a low bacterial load. As the quantity of bacteria in the solution decreases, the ratio of nanoparticles to bacteria increases. This means that more nanoparticles are available to bind and self-assemble on the surface of a bacterium. As the population of bacteria in the solution increases, the resulting higher number of bacteria competes with the available number of nanoparticles in the solution. A nanoparticle-based bacterial detection method would exhibit exceptional sensitivity at low cell densities [27]. Our result is consistent with the work done by Gordillo-Marroquín and others [27], who used a magnetic nanoparticle-based colorimetric biosensing assay (NCBA) to detect pulmonary tuberculosis (PTB) in sputum samples. Their data revealed that the CE decreases linearly with increasing bacterial concentrations, from 95% at 10^1^ CFU/mL to 80% at 10^5^ CFU/mL.

The MNPs captured *Salmonella* up to 1 CFU/mL, showing that MNP is an efficient tool for detecting and isolating foodborne pathogens. Ueda S. et al. [28] used immunomagnetic separation (IMS) to detect *Salmonella* from food and fecal samples contaminated artificially at 1 to 10^3^ CFU/mL and then detected *Salmonella* by PCR assay. With IMS, the authors isolated up to 1 CFU/mL of *Salmonella* from the samples. Although our results are comparable to the work done by these authors, they used the IMS method, which is more expensive than the glycan-coated MNPs used in this study. While the glycan-coated MNPs are non-specific, plating on the selective agar allows for the differentiation of target pathogens. Additionally, the specificity can be enhanced by applying specific carbohydrate epitopes; the main benefit of carbohydrate-functionalized MNPs is their affordability [29]. In one study, for example, the testing cost decreased from USD 0.40 to USD 0.10 per assay using glycan-coated MNPs instead of a comparable antibody-based assay [23]. Compared to IMS, glycan-coated MNPs also require less handling and have a longer shelf life at room temperature, further lowering overall costs [30]. The CE reported in this study was higher than other studies [31,32,33,34], which recorded CEs of 73–90%, 80–88%, 17–34%, and >90%, respectively. Please see the various comparisons summarized in Table 5. For their experiments, these authors employed a variety of glycans as MNP coatings, such as mannose, galactose, and chitosan. These variations could be due to further modifications of the glycan-coated MNPs, such as adding amino acids, which could potentially increase the positive charge of the MNP coating and improve bacterial adhesion [29]. The MNPs used in this study were coated with chitosan, a glycan molecule. The coating is conducted through the chemisorption method. The biosensor response is more associated with the bacterial surface proteins, not the MNPs. Chitosan is appropriate for pathogen adsorption and pathogen identification and is not hampered by other molecules other than the bacteria. A previous study at Dr Alocilja’s lab has shown that the MNPs surface is stable and active for at least three years under room temperature storage [20,22,23,24,25,29,35].

The GNPs biosensor detected *Salmonella* Typhimurium DNA to a 2.9 µg/µL concentration at an equivalent of 5.3 × 10^5^ CFU. Our result is comparable to Vetrone Huarng [23], with an LOD ranging from 1 to 100 ng/mL for *S.* Enteritidis in milk and orange juice samples. Ying et al. [36], however, reported a higher LOD. They used streptavidin (SA)-functionalized gold nanoparticles (GNPs-SA) in lateral flow nano-biosensors. Two sequences were used to increase the specificity, and the detection limit for *Salmonella* was 3 × 10^3^ CFU/mL [36]. This implies that the GNPs biosensor used in this study is sensitive and can potentially be used when a sample’s pathogen load is low. Duan and others [37] used a surface-enhanced Raman spectroscopy (SERS)-based aptasensor, with Au@Ag core/shell nanoparticles as the recognition element for *S*. Typhimurium in an actual food sample, where they successfully detected the pathogen with a detection limit of 15 CFU/mL [37]. In the current study, the GNPs specifically detected *Salmonella* Typhimurium in bovine fecal samples. This is because the probes on the GNP biosensors are relatively long and have a specific complementary DNA to the target pathogen. As a result, there was no cross-reactivity among the four pathogens. As seen in Figure 5a,b, no cross-reactivity was observed when various DNAs were analyzed in complex sample matrices.

The GNPs biosensor was tested on thirty-eight naturally contaminated bovine fecal samples (Table 4). DNA was extracted from these samples and used to test the performance of the GNPs biosensor. Out of the thirty-eight fecal samples tested, 81.6% (31/38) were positive for *Salmonella enterica* spp., 65.8% (25/38) for *C. jejuni*, 55.3% (21/38) for *L. monocytogenes,* and 50% (19/38) for *E. coli* O157:H7. This further validates the efficiency of the GNPs biosensor as an effective tool in detecting foodborne pathogens in a complex matrix (fecal samples). Also, the results suggest that these foodborne pathogens are present and constantly shedding in most Alabama cattle ranches.

The utilization of MNPs, on the other hand, resulted in the detection of the four priority pathogens in fecal samples at a concentration as low as 1 CFU/mL. In contrast, no colony growth was observed in the absence of MNPs. This implies that, due to the tiny quantity of pathogens in some samples, positive cases of foodborne pathogens may go undetected using traditional culture methods. Unfortunately, even a small amount of these pathogens may render the food hazardous if left undetected. In some countries, the infectious dose of *L. monocytogenes*, for example, ranges between 10 and 100 million CFU in healthy hosts and between 0.1 and 10 million CFU in high-risk individuals [38]. However, U.S. regulatory authorities have established zero-tolerance policies regarding *Salmonella enterica* spp. and *L. monocytogenes* in ready-to-eat foods [39]. MNPs capturing 1 CFU/mL will provide an early and efficient diagnosis of foodborne pathogens, preventing their spread. When samples are of a low contamination and infective dose, an excellent detection sensitivity necessitates accurately extracting and concentrating bacteria from complicated matrices [31].

Molecular techniques, such as polymerase chain reaction (PCR), can be utilized to diagnose *Salmonella*. Nevertheless, although these assays exhibit quantitative and high-sensitivity characteristics, they are unsuitable for point-of-care applications due to their cost and time constraints. Concerning this matter, the proposed methodology presented numerous benefits compared to existing methods. The GNPs biosensor described in this study offers a versatile platform for analyzing crude matrices like bovine fecal samples. The traditional surveillance process for *Salmonella* is a multi-step procedure that typically spans five days, starting from sample preparation on day one and concluding with isolate confirmation on day five [40].

**Table 5:** Different MNPs and GNPs for extraction and detection of foodborne pathogens from other studies.

Coating	Bacteria	Matrix	Capture Efficiency	Detection Method	LOD	Reference
Glycan-coated MNPs (chitosan)	*E. coli* O157:H7, *Salmonella enterica* spp., *C. jejuni*, and *L. monocytogenes*	Bovine fecal sample	99–100%	Magnetic nanoparticles	1 CFU/mL	This study
Dextrin-capped GNPs	*E. coli* O157:H7, *Salmonella enterica* spp., *C. jejuni*, and *L. monocytogenes*	Bovine fecal sample	N/A	Plasmonic/colorimetric	2.9 µg/µL, which is 5.3 × 10^5^ cells	This study
Glycan (not specified), cysteine–glycan	*S.* Enteritidis, *E. coli* O157:H7, *B. cereus*	Milk	73–90%	N/A	N/A	[31]
Mannose and galactose	*E. coli*	PBS	80–88%	BacTiter-Glo assay	N/A	[34]
Biotinylated mono- and biantennary di-/trisaccharide	*E. coli* (UPEC)	PBS	17–34%	BacTiter-Glo™ assay	N/A	[32]
Lysine-SCGs	*E. coli* O157:H7	Sausage	>90%	Colorimetric	30.8 CFU/mL	[33]
gold@platinum nanocatalyst (Au@PtNCs)	*Salmonella*	N/A	N/A	Colorimetric	350 CFU/mL	[41]
Gold nanoparticles and asymmetric PCR	*S.* Typhimurium	Lettuce	N/A	Colorimetric	2.56 CFU/mL	[42]
Urease-induced silver metallization on the surface of gold nanorods (AuNR)	*Salmonella* Choleraesuis	Pasteurized whole milk	N/A	Colorimetric ELISA	1.21 × 10^2^ cfu/mL and 1.21 × 10^1^ cfu/mL	[43]
DNA-functionalized gold nanoparticles	*S. aureus*, *S.* Typhimurium, *S.* Enteritidis	Cheese, chicken, lettuce, omelet, and potato salad	N/A	Plasmon-assisted colorimetric detection	1 CFU/mL	[44]
Oligonucleotide–gold nanoparticles	*Salmonella* spp.	Blueberries and chicken meat	N/A	Optical/colorimetric	<10 CFU/mL	[45]

In comparison, experiments run with GNPs can take up to 30 min to detect the presence of a specific pathogen. For MNPs, capturing pathogens will take about 15 min; with a down-the-line analysis PCR or culture, we should be able to detect a particular pathogen within 1–24 h. Conventional procedures may necessitate a greater number of skilled workers to effectively identify and verify the presence of *Salmonella* in each sample, which makes it labor-intensive. This is unlike nanoparticle technology, whereby a single individual may complete the entire pathogen detection procedure quickly from start to finish. Moreover, this assay is cost-effective, estimated at less than USD 0.01 per reaction. Additionally, the equipment required for this assay is minimal, making it suitable for point-of-care applications. These features make the GNPs biosensor a specific and sensitive tool for point-of-care testing [24].

Integrating DNA with nanomaterials in probes has demonstrated notable enhancements in sensitivity and specificity. Furthermore, this approach frequently enables the detection of bacteria without amplification techniques [23].

## 5. Study Limitation

Although the glycan-coated MNPs are nonspecific in pathogen detection, selectivity in natural samples can be improved by utilizing specific carbohydrate epitopes. An example of this is the utilization of biotinylated oligosaccharides attached to streptavidin-coated magnetic beads. These beads have been employed to capture *E. coli* strains with the pap pilus genotype [32].

## 6. Conclusions

In conclusion, the GNPs biosensor demonstrated a high sensitivity and specificity in detecting *L. monocytogenes*, *Salmonella* spp., *E. coli* O157:H7, and *C. jejuni* in bovine fecal samples, which is a challenging and complicated sample matrix. The MNPs exhibited a significant capture efficiency, suggesting their potential as an effective extraction/concentration technique, serving as an alternative to other concentration methods such as centrifugation or filtration. The findings of this work validate the feasibility of using glycan-coated MNPs and GNPs biosensors in the field to identify foodborne pathogens at an early stage, hence reducing the time and effort required. The utilization of nano-biosensor diagnostic techniques will be extremely valuable in identifying the existence of these four major foodborne pathogens in cattle, which is vital for the farm-to-fork continuum, considering that fecal carriage of these pathogens may contaminate carcasses following slaughter and enter the human food chain.

## Figures and Tables

**Table 1:** Bacterial strains used for study.

Bacteria	Strain
*Salmonella* Typhimurium	ATCC 13311
*Listeria monocytogenes*	ATCC 19117
*Escherichia coli* O157:H7	61593
*Campylobacter jejuni*	ATCC 33560

**Table 2:** Probe biosensors used for study.

Target Gene	Probe Name	Sequence (5′–3′)	Tm	Target Bacteria
*invA*	InvA-F-Biosensor	/5AmMC6/CGC TTC GCC GTT CGC GCG CGG CAT CCG CAT CAA TAA TAC C	72.4 °C	*Salmonella enterica* spp.
	Lmo0733-F-Biosensor	/5AmMC6/TA TAC GGT AGA ATA GGT TAA CTG TCC AGT TCC ATT TTT AAC	60.1 °C	*Listeria monocytogenes*
*yeeS*	0157-1-Biosensor	/5AmMC6/AG TCT TGG TGC TGC TCT GAC ATT TTT GGA CTT AGG TAT AG	64.3 °C	*Escherichia coli* O157:H7
*Cj0415*	Cj0414-1-Biosensor	/5AmMC6/GG ATG GAC TGG AGG TAT AGT GGC TGC AGA GCT TAC TAA AG	62.6 °C	*Campylobacter jejuni*

**Table 3:** Results from thirty-eight fecal samples were tested by GNPs and PCR. (+) indicates positive, and (−) indicates negative.

Sample Number		*E. coli* O157:H7		*L. monocytogenes*		*C. jejuni*		*S. typhimurium*
	GNPs	PCR	GNPs	PCR	GNPs	PCR	GNPs	PCR
125	−	−	−	−	−	−	+	+
139	+	+	+	+	+	−	+	+
152	−	−	+	+	+	+	+	+
158	+	+	+	−	+	+	+	+
167	−	−	+	+	+	+	+	+
168	−	−	+	+	+	+	+	+
174	+	−	+	−	+	+	+	+
176	+	−	+	−	+	+	+	+
180	+	+	+	−	−	−	+	+
182	−	−	+	−	+	+	+	+
185	−	−	+	−	−	−	+	+
186	−	+	+	−	−	−	+	+
190	−	−	+	+	+	+	+	+
191	+	+	+	−	+	+	+	+
192	+	+	+	−	+	+	+	+
196	−	−	+	+	−	−	+	+
198	−	+	−	−	−	−	+	+
208	+	+	−	−	−	+	+	+
219	+	+	−	−	+	+	+	+
222	+	+	−	−	−	−	+	+
225	+	+	−	−	+	−	+	+
226	−	−	−	−	+	+	+	+
227	+	+	+	+	+	+	+	+
238	+	+	−	−	+	+	+	+
244	−	−	−	−	+	+	+	+
245	−	−	−	−	+	+	+	+
251	−	+	−	−	−	−	+	+
260	−	−	−	−	−	−	+	+
261	+	+	+	+	−	−	+	+
262	+	+	−	−	+	+	+	+
273	−	−	−	−	+	+	−	−
274	+	+	+	+	−	−	+	+
275	+	+	+	+	−	−	−	−
276	−	−	+	+	+	−	−	−
277	+	−	−	−	+	+	−	−
278	+	+	−	−	+	+	−	−
279	−	−	−	−	+	+	−	−
280	−	−	+	+	+	+	−	−

**Table 4:** Thirty-eight bovine fecal samples tested. GNPs biosensor compared to PCR.

	GNPs +	GNPs −	PCR +	PCR −
*S.* Typhimurium	31	7	31	7
*E. coli* O157:H7	19	19	19	19
*C. jejuni*	25	13	23	15
*L. monocytogenes*	21	17	12	26

## Data Availability

The original contributions presented in the study are included in the article, further inquiries can be directed to the corresponding author.

## References

[1] Heredia N., García S. (2018). Animals as sources of foodborne pathogens: A review. Anim. Nutr..

[2] Zhao X., Lin C.W., Wang J., Oh D.H. (2014). Advances in rapid detection methods for foodborne pathogens. J. Microbiol. Biotechnol..

[3] (2017). European Food Safety Authority. The European Union summary report on trends and sources of zoonoses, zoonotic agents, and foodborne outbreaks in 2016. EFSA J..

[4] Tack D.M., Marder E.P., Griffin P., Cieslak P.R., Dunn J., Hurd S., Scallan E., Lathrop S., Muse A., Ryan P. (2019). Preliminary incidence and trends of infections with pathogens transmitted commonly through food—Foodborne Diseases Active Surveillance Network, 10 US Sites, 2015–2018. Morb. Mortal. Wkly. Rep..

[5] Schneider G., Schweitzer B., Steinbach A., Pertics B.Z., Cox A., Kőrösi L. (2021). Antimicrobial Efficacy and Spectrum of Phosphorous-Fluorine Co-Doped TiO2 Nanoparticles on the Foodborne Pathogenic Bacteria Campylobacter jejuni, Salmonella Typhimurium, Enterohaemorrhagic E. coli, Yersinia enterocolitica, Shewanella putrefaciens, Listeria monocytogenes and Staphylococcus aureus. Foods.

[6] World Health Organization Food Safety 2022. https://www.who.int/news-room/fact-sheets/detail/food-safety.

[7] Mi F., Guan M., Hu C., Peng F., Sun S., Wang X. (2021). Application of lectin-based biosensor technology in the detection of foodborne pathogenic bacteria: A review. Analyst.

[8] Dumen E., Ekici G., Ergin S., Bayrakal G.M. (2020). Presence of foodborne pathogens in seafood and risk ranking for pathogens. Foodborne Pathog. Dis..

[9] Park S.H., Aydin M., Khatiwara A., Dolan M.C., Gilmore D.F., Bouldin J.L., Ahn S., Ricke S.C. (2014). Current and emerging technologies for rapid detection and characterization of Salmonella in poultry and poultry products. Food Microbiol..

[10] Duan N., Xu B., Wu S., Wang Z. (2016). Magnetic Nanoparticles-based Aptasensor Using Gold Nanoparticles as Colorimetric Probes for the Detection of Salmonella typhimurium. Anal. Sci..

[11] Juncker D., Bergeron S., Laforte V., Li H. (2014). Cross-reactivity in antibody microarrays and multiplexed sandwich assays: Shedding light on the dark side of multiplexing. Curr. Opin. Chem. Biol..

[12] Shen Y., Xu L., Li Y. (2021). Biosensors for rapid detection of Salmonella in food: A review. Compr. Rev. Food Sci. Food Saf..

[13] Zhu C., Yang G., Li H., Du D., Lin Y. (2015). Electrochemical sensors and biosensors based on nanomaterials and nanostructures. Anal. Chem..

[14] Jiang C., Lan L., Yao Y., Zhao F., Ping J. (2018). Recent progress in application of nanomaterial-enabled biosensors for ochratoxin A detection. TrAC Trends Anal. Chem..

[15] Jazayeri M.H., Aghaie T., Avan A., Vatankhah A., Ghaffari M.R.S. (2018). Colorimetric detection based on gold nanoparticles (GNPs): An easy, fast, inexpensive, low-cost and short time method in detection of analytes (protein, DNA, and ion). Sens. Bio-Sens. Res..

[16] Khansili N., Rattu G., Krishna P.M. (2018). Label-free optical biosensors for food and biological sensor applications. Sens. Actuators B Chem..

[17] Pérez-López B., Merkoçi A. (2011). Nanomaterials based biosensors for food analysis applications. Trends Food Sci. Technol..

[18] Chen J., Park B. (2016). Recent Advancements in Nanobioassays and Nanobiosensors for Foodborne Pathogenic Bacteria Detection. J. Food Prot..

[19] Deng H., Zhang X., Kumar A., Zou G., Zhang X., Liang X.J. (2013). Long genomic DNA amplicons adsorption onto unmodified gold nanoparticles for colorimetric detection of Bacillus anthracis. Chem. Commun..

[20] Dester E., Kao K., Alocilja E.C. (2022). Detection of Unamplified E. *coli O*157 DNA Extracted from Large Food Samples Using a Gold Nanoparticle Colorimetric Biosensor. Biosensors.

[21] Hung Y.L., Hsiung T.M., Chen Y.Y., Huang Y.F., Huang C.C. (2010). Colorimetric detection of heavy metal ions using label-free gold nanoparticles and alkanethiols. J. Phys. Chem. C.

[22] Lim D., Villame R.G., Quiñones G.J., de Vera D., Notorio R., Fernando L., Alocilja E. (2017). Alocilja Magnetic Nanoparticles Efficiently Capture Escherichia coli O157: H7 Isolates. PJP.

[23] Vetrone S.A., Huarng M.C., Alocilja E.C. (2012). Detection of non-PCR amplified *S. enteritidis* genomic DNA from food matrices using a gold-nanoparticle DNA biosensor: A proof-of-concept study. Sensors.

[24] Baetsen-Young A.M., Vasher M., Matta L.L., Colgan P., Alocilja E.C., Day B. (2018). Direct colorimetric detection of unamplified pathogen DNA by dextrin-capped gold nanoparticles. Biosens. Bioelectron..

[25] Bhusal N., Shrestha S., Pote N., Alocilja E.C. (2018). Nanoparticle-Based Biosensing of Tuberculosis, an Affordable and Practical Alternative to Current Methods. Biosensors.

[26] Mooijman K.A., Pielaat A., Kuijpers A.F. (2019). Validation of EN ISO 6579-1-Microbiology of the food chain-Horizontal method for the detection, enumeration and serotyping of Salmonella-Part 1 detection of *Salmonella* spp.. Int. J. Food Microbiol.

[27] Gordillo-Marroquín C., Gómez-Velasco A., Sánchez-Pérez H.J., Pryg K., Shinners J., Murray N., Muñoz-Jiménez S.G., Bencomo-Alerm A., Gómez-Bustamante A., Jonapá-Gómez L. (2018). Magnetic nanoparticle-based biosensing assay quantitatively enhances acid-fast bacilli count in paucibacillary pulmonary tuberculosis. Biosensors.

[28] Ueda S., Umesako S., Mineno J., Kuwabara Y. (2000). The Magnetic Immuno Polymerase Chain Reaction Assay for Detection of Salmonella from Food and Fecal Samples. Biocontrol Sci..

[29] Dester E., Alocilja E. (2022). Current Methods for Extraction and Concentration of Foodborne Bacteria with Glycan-Coated Magnetic Nanoparticles: A Review. Biosensors.

[30] Matta L.L. (2018). Biosensing Total Bacterial Load in Liquid Matrices to Improve Food Supply Chain Safety Using Carbohydrate-Functionalized Magnetic Nanoparticles for Cell Capture and Gold Nanoparticles for Signaling. Ph.D. Dissertation.

[31] Matta L.L., Alocilja E.C. (2018). Carbohydrate Ligands on Magnetic Nanoparticles for Centrifuge-Free Extraction of Pathogenic Contaminants in Pasteurized Milk. J. Food Prot..

[32] Yosief H.O., Weiss A.A., Iyer S.S. (2013). Capture of uropathogenic E. coli by using synthetic glycan ligands specific for the pap-pilus. Chembiochem A Eur. J. Chem. Biol..

[33] You S.M., Jeong K.B., Luo K., Park J.S., Park J.W., Kim Y.R. (2021). based colorimetric detection of pathogenic bacteria in food through magnetic separation and enzyme-mediated signal amplification on paper disc. Anal. Chim. Acta..

[34] El-Boubbou K., Gruden C., Huang X. (2007). Magnetic glyco-nanoparticles: A unique tool for rapid pathogen detection, decontamination, and strain differentiation. J. Am. Chem. Soc..

[35] Matta L.L., Harrison J., Deol G.S., Alocilja E.C. (2018). Carbohydrate-functionalized nanobiosensor for rapid extraction of pathogenic bacteria directly from complex liquids with quick detection using cyclic voltammetry. IEEE Trans. Nanotechnol..

[36] Ying N., Ju C., Li Z., Liu W., Wan J. (2017). Visual detection of nucleic acids based on lateral flow biosensor and hybridization chain reaction amplification. Talanta.

[37] Duan N., Chang B., Zhang H., Wang Z., Wu S. (2016). Salmonella typhimurium detection using a surface-enhanced Raman scattering-based aptasensor. Int. J. Food Microbiol..

[38] Farber J., Ross W., Harwig J. (1996). Health risk assessment of Listeria monocytogenes in Canada. Int. J. Food Microbiol..

[39] Mendonça M., Bhunia A. (2015). Fiber-Optic Sensors for High Throughput Screening of Pathogens.

[40] United States Department of Agriculture Food Safety and Inspection Service MLG 4.13 Isolation and Identification of Salmonella from Meat, Poultry, Pasteurized Egg, Carcass, and Environmental Sponges 2023. https://www.fsis.usda.gov/sites/default/files/media_file/documents/MLG-4.13.pdf.

[41] Qi W., Zheng L., Hou Y., Duan H., Wang L., Wang S., Liu Y., Li Y., Liao M., Lin J. (2022). A finger-actuated microfluidic biosensor for colorimetric detection of foodborne pathogens. Food Chem..

[42] Wang L., Wu X., Hu H., Huang Y., Yang X., Wang Q., Chen X. (2021). Improving the detection limit of Salmonella colorimetry using long ssDNA of asymmetric-PCR and non-functionalized AuNPs. Anal. Biochem..

[43] Gao B., Chen X., Huang X., Pei K., Xiong Y., Wu Y., Duan H., Lai W., Xiong Y. (2019). Urease-induced metallization of gold nanorods for the sensitive detection of Salmonella enterica Choleraesuis through colorimetric ELISA. J. Dairy Sci..

[44] Sanromán-Iglesias M., Garrido V., Gil-Ramírez Y., Aizpurua J., Grzelczak M., Grilló M.J. (2021). Plasmon-assisted fast colorimetric detection of bacterial nucleases in food samples. Sens. Actuators B Chem..

[45] Quintela I.A., de Los Reyes B.G., Lin C.S., Wu V.C. (2019). Simultaneous colorimetric detection of a variety of Salmonella spp. in food and environmental samples by optical biosensing using oligonucleotide-gold nanoparticles. Front. Microbiol..

